# Spns1 is an iron transporter essential for megalin-dependent endocytosis

**DOI:** 10.1152/ajprenal.00172.2024

**Published:** 2024-09-12

**Authors:** Andrew Beenken, Tian Shen, Guangchun Jin, Aryan Ghotra, Katherine Xu, Kivanc Nesanir, Rachel E. Sturley, Soundarapandian Vijayakumar, Atlas Khan, Abraham Levitman, Jacob Stauber, Estefania Y. Chavez, Shelief Y. Robbins-Juarez, Luke Hao, Thomas B. Field, Hediye Erdjument-Bromage, Thomas A. Neubert, Lawrence Shapiro, Andong Qiu, Jonathan Barasch

**Affiliations:** ^1^Division of Nephrology, Department of Medicine, Columbia University Vagelos College of Physicians and Surgeons, New York, New York, United States; ^2^Columbia College, Columbia University, New York, New York, United States; ^3^University of Richmond, Richmond, Virginia, United States; ^4^Albert Einstein College of Medicine, New York, New York, United States; ^5^Department of Pediatrics, Columbia University Vagelos College of Physicians and Surgeons, New York, New York, United States; ^6^Department of Medicine, Duke University Medical Center, Durham, North Carolina, United States; ^7^Department of Mechanical Engineering, University of Vermont, Burlington, Vermont, United States; ^8^Department of Neuroscience and Physiology and Neuroscience Institute, New York University Grossman School of Medicine, New York, New York, United States; ^9^Zuckerman Mind Brain Behavior Institute, Columbia University, New York, New York, United States; ^10^Aaron Diamond AIDS Research Center, Columbia University, New York, New York, United States; ^11^Department of Biochemistry and Molecular Biophysics, Columbia University, New York, New York, United States; ^12^Advanced Institute of Translational Medicine, Tongji University, Shanghai, China; ^13^Department of Pathology and Cell Biology, Columbia University, New York, New York, United States; ^14^Columbia University George M. O’Brien Urology Center, New York, New York, United States

**Keywords:** endocytosis, iron, megalin, proteinuria, Spns1

## Abstract

Proximal tubule endocytosis is essential to produce protein-free urine as well as to regulate system-wide metabolic pathways, such as the activation of Vitamin D. We have determined that the proximal tubule expresses an endolysosomal membrane protein, protein spinster homolog1 (Spns1), which engenders a novel iron conductance that is indispensable during embryonic development. Conditional knockout of Spns1 with a novel Cre-LoxP construct specific to megalin-expressing cells led to the arrest of megalin receptor-mediated endocytosis as well as dextran pinocytosis in proximal tubules. The endocytic defect was accompanied by changes in megalin phosphorylation as well as enlargement of lysosomes, confirming previous findings in Drosophila and Zebrafish. The endocytic defect was also accompanied by iron overload in proximal tubules. Remarkably, iron levels regulated the Spns1 phenotypes because feeding an iron-deficient diet or mating Spns1 knockout with divalent metal transporter1 knockout rescued the phenotypes. Conversely, iron-loading wild-type mice reproduced the endocytic defect. These data demonstrate a reversible, negative feedback for apical endocytosis and raise the possibility that regulation of endocytosis, pinocytosis, megalin activation, and organellar size and function is nutrient-responsive.

**NEW & NOTEWORTHY** Spns1 mediates a novel iron conductance essential during embryogenesis. Spns1 knockout leads to endocytic and lysosomal defects, accompanied by iron overload in the kidney. Reversal of iron overload by restricting dietary iron or by concurrent knockout of the iron transporter, DMT1 rescued the endocytic and organellar defects and reverted markers of iron overload. These data suggest feedback between iron and proximal tubule endocytosis.

## Graphical abstract

**Figure:**
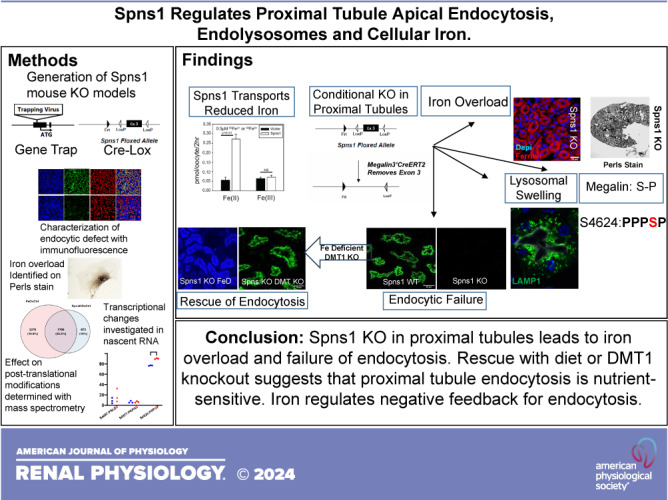

## INTRODUCTION

The recovery of electrolytes (Na^+^, K^+^, Ca^2+^, Mg^2+^, and PO_4_^2-)^ and water from the glomerular filtrate by different segments of the kidney tubule has been detailed over the past half-century. However, the mechanisms that recover filtered proteins have remained obscure. Megalin (*Lrp2*) and its partner cubilin are thought to bind and transport as much as ∼2 g/day of low molecular weight (LMW) proteins that escape glomerular barriers (1). Protein endocytosis is a central function of the proximal tubule, not only producing protein-free urine but also capturing metabolic precursors for metabolism in proximal cells, such as the precursor of active vitamin D.

Some of the filtered proteins contain iron; the disposition of their iron is complicated because different iron species with differing physical properties require different transport carriers. The common, oxidized form of iron (Fe^3+^) precipitates at pH > 4, and thus dedicated carriers are required to solubilize Fe^3+^ and conduct it into cells. Transferrin-Fe^3+^ is the most prominent iron transport protein (2–4), releasing Fe^3+^ in endosomes at low pH, while apotransferrin recycles to the plasma membrane by remaining bound to its receptor transferrin-TFR1 (5, 6). Endosomal iron can be exported to the cytoplasm by DMT1 after the reduction of Fe^3+^ to Fe^2+^ by ferrireductases (7). Iron can also enter the proximal tubule via megalin-cubilin rather than TFR1 due to apical capture of transferrin and LMW proteins associated with iron.

TFR1 and DMT1 are ubiquitous and conserved among species, but their deletions produce tissue- and stage-specific phenotypes rather than global defects, implicating additional transporters (8–14). Using distant homology blast searches with known iron transporter sequences as bait, we identified *Spns1* as a candidate iron transporter. We report that global deletion of *Spns1* is embryonic lethal while conditional knockout in the kidney proximal tubule generates cytoplasmic iron overload and near complete suppression of apical endocytosis. These phenotypes are linked because dietary iron restriction or concurrent conditional knockout of DMT1 restores proximal tubular function. Conversely, dietary iron overload mimics the deletion of *Spns1*. Given the preserved expression of megalin in *Spns1* conditional knockout, this study is hypothesis generating as to possible posttranslational regulation of megalin and, more generally, to feedback from the ligand to the endocytic process that captured the ligand. Our results provide evidence that proximal tubule cells are responsive to metabolic stimuli and sensitive to negative feedback from the ligands they capture.

These data indicate a novel second endosomal-lysosomal function of *Spns1*, which is known to export lysophosphatidyl choline and lysophosphatidyl ethanolamine from endosome and lysosomes and supply these lipids to the cytoplasm (15–17).

## MATERIALS AND METHODS

### Database Mining of the Mouse Genome

Using the search parameter of Distant Homology, the Ensembl human peptide database was blasted with conserved domains of two SLC11 family members (GenBank Accession No. pfam01770.12) and yeast genes *Fet4* (a low-affinity Fe^2+^ transporter) and *Ftr1* (a high-affinity membrane conductance specific to Fe^3+^). The search produced a series of distant homologs for downstream tests.

### Uptake Studies in *Xenopus* Oocytes

The open reading frames of candidate genes were PCR amplified from E15 mouse embryo cDNA and then cloned into pSPT64T or pTNii plasmids for the synthesis of capped sense cRNA from the SP6 promoter using the mMESSAGE mMACHINE system (AM1344, Ambion, Austin, TX). Defolliculated *Xenopus laevis* oocytes were prepared as described (18) and injected with 50 nL of water or cRNA of candidate genes (30 ng). Radiotracer uptake was determined 3 or 4 days later. Seven to ten oocytes were incubated in 500 µL of modified Barth’s solution [EcocyteShop; MBS; 88 mM NaCl, 2.4 mM NaHCO_3_, 2.5 mM Na pyruvate, 1 mM KCl, 0.82 mM MgSO_4_, 0.41 mM CaCl_2_, 0.3 mM Ca(NO_3_)_2_, and 15 mM MES or HEPES], and uptake of ^55^Fe^2+^ (1 mM ascorbate; Fe:NTA = 1:4) or ^55^Fe^3+^ (Fe:NTA = 1:4) was assessed at room temperature. Uptake was halted by the addition of ice-cold MBS containing 100 mM nonradiolabeled FeCl_3_ and 1 M ascorbate (pH 7.5). Oocytes were washed 10 times thereafter and solubilized with 10% SDS for measurement of radioactivity.

### Mice

Mouse breeding, feeding with control and iron**-**deficient diets, and euthanasia followed protocols approved by the Columbia Institutional Animal Care and Use Committee (IACUC). Equal responses in male-female mice and female C57Bl6 mice were studied.

### Generation of Spns1-Inactivated Mice

We obtained the *Spns1*-trapped embryos (2–4 cell stages, C57BL/6;129S5) from Mouse Mutant Regional Resource Centers (MMRRC_011643-UNC), which were then resuscitated at Columbia. One allele of the *Spns1* gene was trapped by a retroviral vector (pUPATrap-CRV2) in the first exon just ahead of the start codon, ATG. The heterozygotes (*Spns1^trap/+^*) were crossed with wild-type C57BL/6 mice for five generations and subsequently bred to homozygosity. The gene trapping-mediated Spns1 ablation (termed *Spns*^−/−^) was verified by quantitative RT-PCR and then PCR genotyped.

### Generation of* Spns1^**+/**nLacZ ^*Mice and *Spns1^flox/^^flox ^*Mice

**Figure 1.**
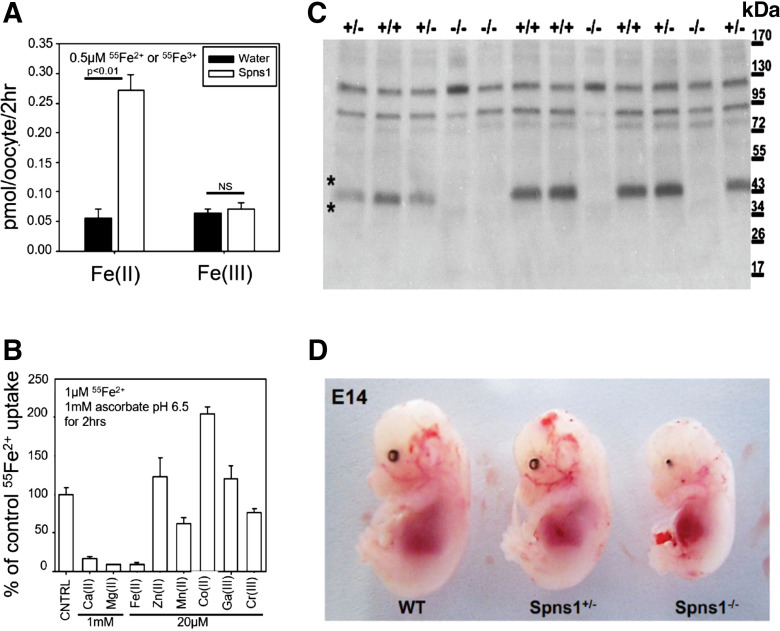
Spns1 is an iron transporter essential for organogenesis. *A*: oocytes inoculated with *Spns1* message transport ^55^Fe^2+^ but not Fe^3+^ (7–10 oocytes/assay; two-tailed *t* test with Welch’s correction). *B*: Fe^2+^, Ca^2+^, and Mg^2+^ competed for ^55^Fe^2+^ capture (7–10 oocytes/assay; *P* < 0.01). *C*: loss of SPNS1 protein in *Spns1*^−/−^ mice. In the immunoblot, the ∼50 kDa SPNS1 band is bracketed by asterisks (*). *D*: failure of organogenesis in gene-trapped *Spns1* knockout mice. Eye development and dorsal cranial structures were most affected.

Blastocysts were inoculated with *Spns1*-targeted ES cells from https://www.informatics.jax.org/allele/MGI:4432137. The targeting cassette was inserted at position 125974001 of chromosome 7 upstream of exon 3. The cassette was composed of an *Frt* site followed by L*acZ* sequence and a *loxP* site followed by a neomycin resistance gene under the control of the human β-actin promoter. A SV40 polyA sequence and a second *Frt* site and second loxP site followed. A third loxP site was inserted downstream of exon 3 at position 125974818. Chimeras were bred with C57BL/6 mice for germline passage. Heterozygous mice were bred with *E2a-Cre-* to generate a *Spns1KO-LacZ* reporter allele or with *Actin-Flipase* to generate a *Spns1* floxed allele (*Spns1^flox/+^*) (Fig. 1).

### Generation of* Megalin3’Cre-Ert*,* Megalin3’Cre-Ert;Spns1^flox/flox^;Uprt^flox/+^*, and *Megalin3’Cre-Ert Spns1^flox/+^;Uprt^flox/+^* Mice

The generation of these mice has been previously described in our published work (19). We used BAC recombineering to remove megalin’s stop codon and replace it with *“P2a-Cre-Ert2-frt-neo-frt*.” The BAC clone was validated by sequencing and by 5' and 3' junctional PCR. The targeted ES cells were confirmed by sequencing and PCR, and, finally, the engineered mice were crossed with *Actin-Flpe* mice to remove the Neo cassette. “P2a” permits the expression of native megalin and *3’Cre-Ert2* from the megalin promoter. We used this Cre driver to delete *Spns1* in the megalin domain without affecting the transcription of megalin itself. Hence, *Megalin3’Cre-Ert;Spns1^flox/flox^* deletes SPNS1 in proximal tubules, but megalin expression is preserved.

Including one allele of promiscuous uracil phosphoribosyl transferase (*Uprt^flox-stop-flox/+^*) resulted in megalin-driven expression of UPRT in the megalin-Cre domain. UPRT incorporates 4-thiouracil in nascent RNA in the proximal tubule in a 4-h window of treatment with 4-thiouracil. Hence, utilizing the *Megalin3’Cre-Ert;Spns1^flox/flox^;Uprt^flox/+^* mice and *Megalin3’Cre-Ert;Spns1^flox/+^;Uprt^flox/+^* mice compare nascent 4-thiouracil labeled RNA in *Spns1^flox/flox^* deleted proximal tubules with nascent labeled RNA in *Spns1^flox/+^* mice depicting time-stamped proximal tubule-specific nascent RNA. Because UPRT incorporates thiouracil, labeled proximal tubule RNA can be isolated from the whole kidney by using thio-biotin-avidin beads. Purification of cell-specific nascent RNA emphasizes the transcriptional response to the knockout (19). All mouse pairs, including *Megalin3’Cre-Ert* and *Spns1^flox/flox^* and *Spns1^flox/+^*, were inoculated with tamoxifen in parallel.

### Measurement of Nascent Proximal Tubule mRNA (Uprt-RNAseq)

Protocols and statistical analyses were conducted as previously published by our laboratory (19). In brief, nascent RNA in the proximal tubule was labeled with 4-thio-uracil and captured with thio-biotin-avidin beads, and 20–60 ng of purified biotinylated RNA were submitted to the Columbia University Genome Center for RNA sequencing. Libraries were prepared using paired-end 100 bp reads for each sample with Illumina TruSeq RNA prep kits and sequenced using Illumina NovaSeq 6000—40 million reads.

### Data Analysis

Illumina RTA was used to perform base calling and Kallisto (version 1.8.2) was used for converting base call files (.BCL) to FASTQ and for performing sequence adaptor trimming. Reads were then trimmed using Trimmomatic version 0.36 (LEADING: 10 TRAILING: 10 MINLEN:30) and then aligned to the mouse reference genome (mm10) using STAR aligner version 2.5.3a (default settings). Count tables were generated with HTSeq (version 0.6.1p2). Gene expression was normalized to transcript length and library size (TPM). Data were deposited in the Gene Expression Omnibus (series GSE268837).

Raw count data across all samples were used to generate PCA plots and differential gene expression using DESeq2 in the Bioconductor package (R version 3.3.2). We curated differentially expressed genes (DEGs) up- or downregulated (*P*_adj_ < 0.05 and >0.5 or <−0.5 log_2_FC) in *Megalin3’Cre-Ert;Spns1^flox/flox^;Uprt^flox/+^* mouse kidneys (“Spns1 KO”) and iron-overloaded *Megalin3’Cre-Ert;Spns1^flox/+^;Uprt^flox/+^* mouse kidneys (“FeO”) compared with *Megalin3’Cre-Ert;Spns1^flox/+^;Uprt^flox/+^* mouse kidneys (“WT”) from littermates (Supplemental Table S2). Venn diagrams were created using the VennDiagram package in R. Heatmaps were created using pHeatmap in R.

Counts from all expressed genes, base count > 0, were normalized by DESeq2 and used to identify enriched gene sets via gene set enrichment analysis (GSEA). We used geneset permutation against Hallmark and mp.cp.v2023.2.Mn.symbols.gmt (Reactome, Biocarta, WikiPathways) databases. Significance was based on FDR q-val < 0.05. We curated genesets that were enriched or deenriched in FeO and Spns1 KO compared with WT mouse kidneys from littermates.

### Genotyping of Animals

Spns1 conditional knockouts were genotyped, yielding a 344 bp band for WT and a 378 bp band for the floxed allele. Genotype of *Megalin3’Cre-Ert;DMT1^flox/flox^* conditional knockout yielded a 376 bp band for WT and 450 bp band for the floxed allele (Supplemental Table S4).

### Iron Overload Protocol

Mice are treated with intraperitoneal injection of 12 mg of elemental iron (iron sucrose, Venofer) daily for 3 days and euthanized 1 wk later according to IACUC protocols.

### Iron-Deficient Diet Protocol

According to the IACUC protocol, mice were placed either on a standard diet (PicoLab 5053) containing 220 ppm iron or on an iron-restricted diet (2-6 ppm iron, TD.80396, Harlan) post weaning, followed by serial weekly ophthalmic vein bleeding for 3 wk. In the subsequent week, mice were euthanized, and hematocrit was evaluated with iSTAT-EC8+ cartridges (Abbott Labs).

### Immunohistochemistry

To generate anti-mouse SPNS1 protein, a peptide (RRAQLHVQGLLHESGPSDDR) corresponding to amino acids 490–509 was synthesized and conjugated with KLH. Rabbit antibodies specific for SPNS1 were affinity purified by Open Biosystems. Tissues were perfusion fixed with 2% paraformaldehyde/PBS buffer and then transferred to 30% sucrose/PBS buffer on a shaker at 4°C. After embedding in O.C.T (Tissue-Tek), frozen sections (15 µm) were prepared, permeabilized with 0.1% Triton X-100/0.2% BSA in PBS for 15 min at room temperature, blocked with 3% BSA in PBS-T, and probed with antibodies (Supplemental Table S4).

### Perls/Turnbull Staining/Periodic Acid-Schiff Staining

Mouse embryonic fibroblasts (MEFs) were prepared from *Spns1*-trapped embryos and *Spns1* expression was determined by RT-PCR. MEFs were incubated in MEM with Holo-Tf (100 μg/mL; Sigma, T1283) overnight and prepared for histochemistry according to published protocols (20, 21). Cryosections of the kidney were also prepared. In brief, for Perls stain, sections were fixed with 1% potassium ferrocyanide with 4% paraformaldehyde in 0.9% NaCl/100 mL water/5 mM PO_4_ (pH 0.8–1.0); for Turnbull, sections were fixed with 1% potassium ferricyanide with 4% paraformaldehyde in 0.9% NaCl/100 mL water/5 mM PO_4_ (pH 0.8–1.0). For both stains, methanol (with 0.01 M NaN_3_ and 0.3% H_2_O_2_) postfixation was followed by incubation with 3,3′-diaminobenzidine-4 HCl (0.025%; Pierce, 1856090; Thermo Fisher Scientific, 34065) and H_2_O_2_ (0.005%) in PBS for 10–30 min. For periodic acid–Schiff stain, paraffin slides were deparaffinized and the Sigma 395B kit was used according to the microwave protocol (22).

### Quantification of Lysosomal Density

A confocal Leica SP8-DLSM light sheet microscope was used. The circumference of megalin^+^ tubules was traced in ImageJ using Ezrin costaining. The outside of the region of interest (ROI) was cleared, and the inside of the ROI was “thresholded” using the Bersen method to identify LAMP1^+^ lysosomes. The total area of identified lysosomes was then quantified using the analyze particles tool with minimum size = 0.3 and circularity = 0.0–1.00. The percent lysosomal area was then calculated by dividing the total lysosomal area by the total ROI.

### Western Blotting

Samples of urine from five control (*Spns1^flox/flox^*) and five conditional knockout (*Megalin3’Cre-Ert;Spns1^flox/flox^*) mice were loaded on SDS-PAGE gel. Proteins were transferred onto nitrocellulose membranes using the Bio-Rad Transblot Turbo System, blocked with 1% nonfat dry milk in TBS-T for 1 h, and probed with primary and secondary antibodies (anti-rabbit HRP; 1:10,000; Jackson Laboratory), followed by imaging with a X-M1 Fujifilm camera using the KwikBlot detection system (Kindle BioSciences).

### Apical Membrane Purification and Mass Spectrometry

LC-MS/MS to identify peptides was conducted as previously published by our laboratory (23). For phospho-proteomic analysis, apical membrane preparations from *Spns^flox/^^flox^* and *Megalin3’Cre-Ert;Spns^flox/^^flox^* mice in triplicate were run ∼1 cm into a SDS-PAGE gel and fixed by Coomassie staining. Proteins in the gel slices were digested with mass spectrometry grade trypsin (400 ng, Promega). Peptides were extracted and desalted using hand-packed C18 columns. Peptide pools from each sample were subjected to phosphopeptide enrichment using titansphere titanium dioxide microcolumns (GL Sciences). After washing, bound phosphopeptides were eluted with a high pH buffer. Flow through as well as the elution were individually analyzed with nano LC-MS/MS over a 2-h acquisition time. Two independent mouse protein database search engines were utilized: MaxQuant 1.5.5.1 and MASCOT 2.5.0. MaxQuant identified 39 unique phosphosites using FDR 0.01 and Mascot identified 102 unique phosphosites with a significance threshold *P* < 0.05. All phosphopeptides were detected in enriched fractions from the elution, and all nonphosphorylated corresponding peptides were found in flowthrough fractions. Additional triplicate sets for *Spns^flox/flox^* and *Megalin3’Cre-Ert;Spns^flox/flox^* mice were analyzed, without enrichment, to quantify the three phospho-serine sites at amino acids S4467, S4577, and S4624. Each phosphopeptide was confirmed with MS/MS evidence. Significant abundance differences were determined with a two-tailed *t* test with Welch’s correction.

## RESULTS

### Spns1 Traffics Iron

To identify novel iron transporters in kidney physiology, we created a database based on distant homology blast searches using yeast proteins FET4 (a low-affinity Fe^2+^ transporter) and FTR1 (a high-affinity membrane conductance specific to Fe^3+^) and mammalian DMT1 (SLC11A2) and metal transporter NRAMP1 (SLC11A1) as bait. Twenty-eight unclassified transmembrane proteins and four known divalent metal transporters (SLC41a1, SLC41a2, SLC39A14, and NIPAL2) were identified. The unclassified proteins were screened by expression in oocytes, using the capture of Fe^2+^ or Fe^3+^ to identify iron transport candidates.^55^Fe^2+^ (1 mM ascorbate; Fe:NTA = 1:4; 2 µM) was reproducibly captured by *Spns1* expressing oocytes following Michaelis–Menten kinetics (*K*_m_ = 3.0 ± 1.1 µM; pH 6.5). Iron capture was specific to Fe^2+^ (Fig. 1*A*) and was inhibited by excess Fe^2+^ (20 µM) and by Ca^2+^ and Mg^2+^ (1 mM) but not by other divalent or trivalent metals, including Fe^3+^ (Fig. 1*B*). Hence, *Spns1* facilitates the transport of Fe^2+^.

### Spns1 Is Essential for Organogenesis

*Spns1* encodes a highly conserved 12-transmembrane protein [(mouse vs. human (92.6%) vs. zebrafish (68.3%) vs. *Drosophila* (54%)] (24). Knockout of the *Drosophila* or zebrafish *Spns1* homolog resulted in the accumulation of lipofuscin in an expanded endosomal-lysosomal system in the nerve, ovary, glia, and neuromuscular junction or in the yolk sac, respectively, suggesting that SPNS1 is critical in transfer of endocytic material from the endosome to lysosome (24–28).

To characterize SPNS1 activity in mice (29, 30), we first determined whether *Spns1* was essential in mouse embryogenesis. We used MMRRC founder (RRID: MMRRC 011643-UNC) containing a retroviral insertion just prior to ATG. The mice (*Spns1*^−/−^) demonstrated loss of immunoreactive SPNS1 and a catastrophic phenotype, including a defective skull, cranium eye organogenesis, and embryonic lethality at E14.5 (Fig. 1, *C* and *D*).

### Conditional Knockout of Spns1 Impairs Proximal Tubule Endocytosis and Causes Proteinuria

**Figure 2.**
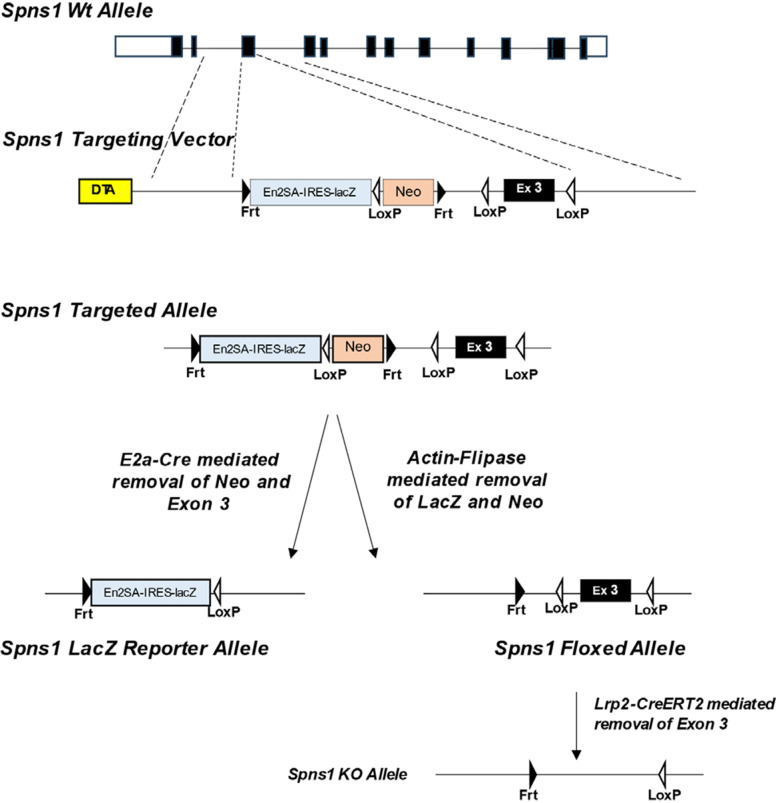
Generation of *Spns1 LacZ* reporter and conditional knockout. We obtained *Spns1*-targeted ES cells from https://www.informatics.jax.org/allele/MGI:4432137. Heterozygotes were bred with *E2a-Cre* to generate *Spns1^LacZ^* reporter mice or bred with *Actin-Flipase* to generate *Spns1^flox/+^* mice, which, in turn, were bred with *MegalinCre-Ert2* mice to obtain *Spns1* deletion specifically in the proximal tubule.

**Figure 3.**
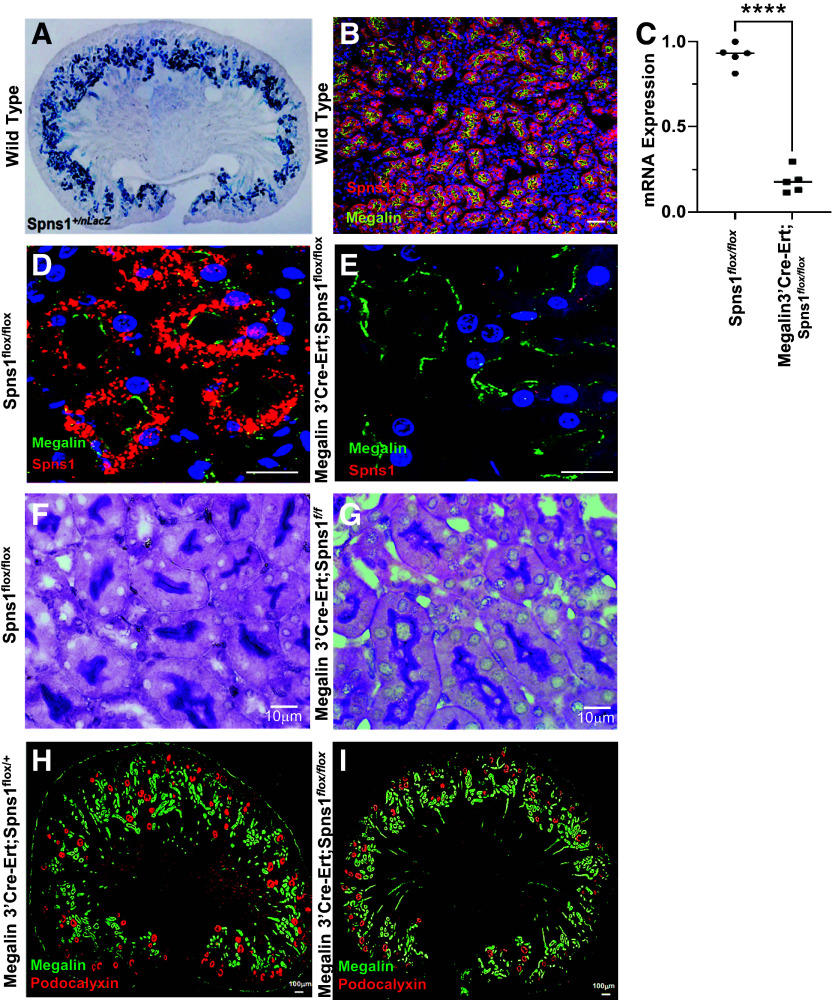
Conditional knockout of *Spns1*. *A*: whole-mount *Spns1^+/Lacz^* in proximal tubules (E13.5). *B*: expression of *Spns1* (red) in megalin^+^ (yellow) proximal tubules. *C*: deletion of *Spns1* expression in *Megalin3’Cre-Ert; Spns1^flox/^^flox^* kidneys (*P* = 1.7 × 10^−7^, two-tailed *t* test with Welch’s correction). *D* and *E*: kidneys from littermates showing with immunofluorescence depicting loss of SPNS1 (red stain) but not megalin (green stain) in *Megalin3’Cre-Ert;Spns1^flox/flox^* knockout proximal tubules (*right*); *Spns1^flox/flox^* is shown as a control (*left*). *F* and *G*: kidneys from littermates with. periodic acid-Schiff staining demonstrating intact apical brush borders in both *Spns1^flox/flox^* controls and *Megalin3’Cre-Ert;Spns1^flox/flox^* knockout proximal tubules. *H* and *I*: kidneys from littermates with immunofluorescence depicting grossly similar levels and distributions of podocalyxin (red stain) and megalin (green stain) in *Megalin3’Cre-Ert;Spns1^flox/^^flox^* knockout proximal tubules (*right*) and *Megalin3’Cre-Ert;Spns1^flox/+^* control (*left*) proximal tubules. Bars = 50 µm in *B*, 20 µm in *D* and *E*, 10 µm in *F* and *G*, and 100 µm in *H* and *I*.

Kidney morphogenesis begins at E11; hence, to investigate *Spns1* function in the kidney, we created two conditional deletions to obviate embryonic lethality and permit investigation of perinatal and adult kidney physiology (Fig. 2). *Spns1^+/LacZ^* revealed prominent *Spns1* driven LacZ in developing E18 cortical tubules with limited expression in other segments (Fig. 3*A*). Consistently, SPNS1 expression colocalized with megalin in the proximal tubule of the adult kidney (Fig. 3*B*).

To determine the role of SPNS1 in the proximal tubule, we used BAC cloning and inserted the *Cre-Ert* driver in place of the megalin stop codon (*Megalin3’Cre-Ert*). This construct permits uninterrupted megalin expression while driving inducible *Cre-Ert* from the megalin promoter (19). *Megalin3’Cre-Ert* efficiently deleted gene expression in megalin-expressing cells. Hence, breeding *Megalin3’Cre-Ert* with *Spns1^flox/flox^* mice deleted proximal tubule *Spns1* RNA (Fig. 3*C*) and protein (Fig. 3, *D* and *E*). Periodic acid–Schiff staining (Fig. 3, *F* and *G*) and immunostains of glomeruli (podocalyxin) and proximal tubules (megalin) (Fig. 3, *H* and *I*) demonstrated morphologically normal kidneys.

**Figure 4.**
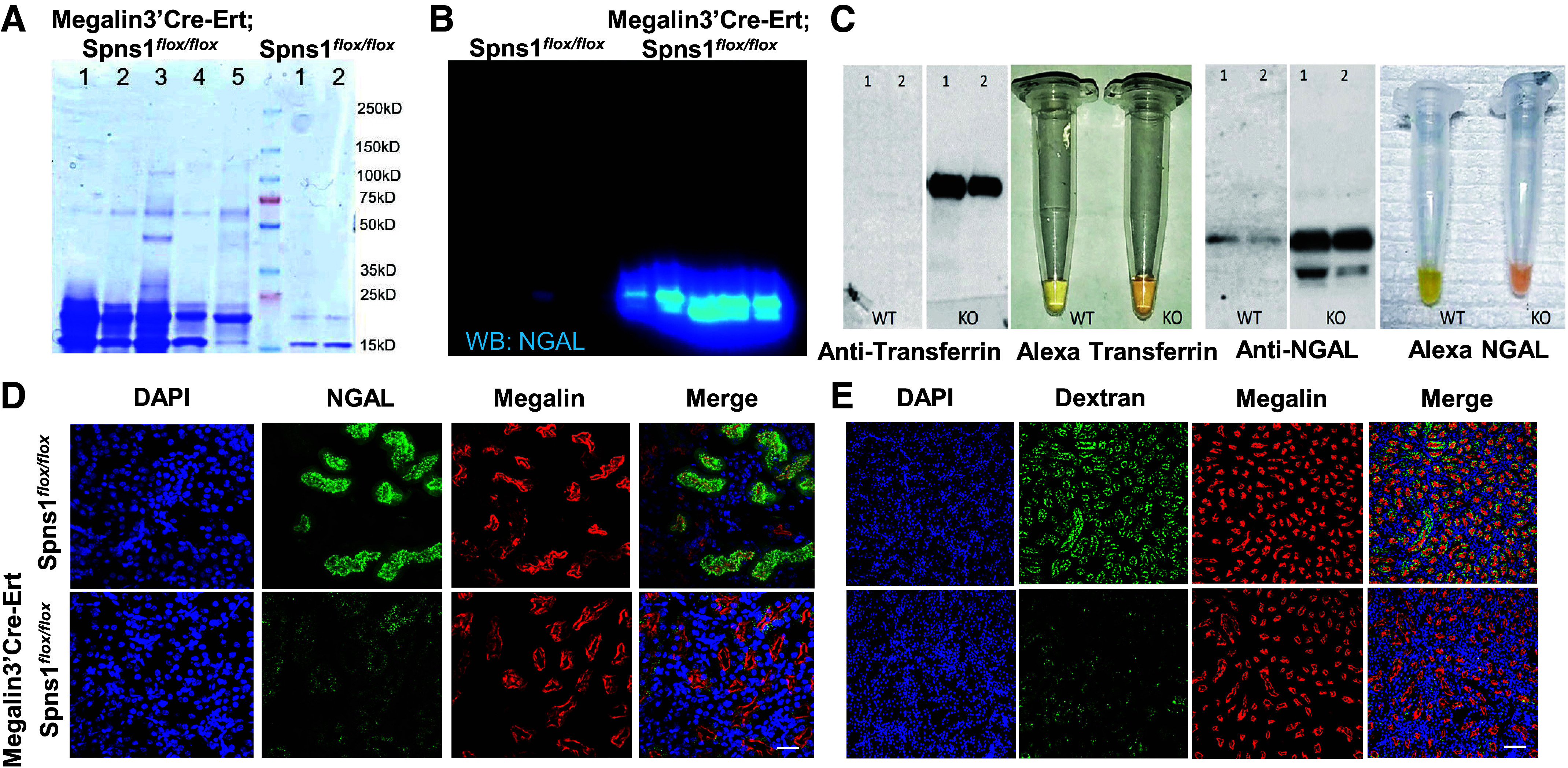
Conditional knockout of Spns1 impairs proximal tubule endocytosis. *A*: low molecular weight proteinuria in *Spns1* knockouts. Coomassie staining is shown. *B*: urinary NGAL excreted in *Spns1* knockouts. *C*: exogenous Alexa568-transferrin and -NGAL excreted in *Spns1* knockout. Urine immunoblots are shown. *D* and *E*: failure to capture FITC-NGAL and FITC-dextran despite expression of megalin in *Megalin3’Cre-Ert;Spns1^flox/flox^* knockout proximal tubules. Bars = 20 µm in *D* and 80 µm in *E*.

The proximal tubule carries out a number of functions essential for systemic homeostasis, including the capture of electrolytes and water and the capture of proteins filtered across glomerular filtration barriers, known as LMW proteins. Consequently, one measure of dysfunction of the proximal tubule is the presence of LMW proteinuria. We examined endogenous proteinuria, and we tested the response of the kidney to the infusion of labeled proteins. We found LMW proteinuria in five *Megalin3’Cre-Ert;Spns1^flox/flox^* mice (Fig. 4*A*). Over 100 urinary proteins were enriched (total spectral counts) compared with control *Spns1^flox/^^flox^* urine (Supplemental Table S1). Serum major urinary proteins (MUP2, MUP3, MUP5, MUP17, MUP18, and MUP20; 21 kDa) (31), trafficking from the liver, predominated in the urine of deleted mice. *Spns1* KO urine also contained endogenous NGAL, which marks proximal and distal tubular damage (Fig. 4*B*) (32).

**Figure 5.**
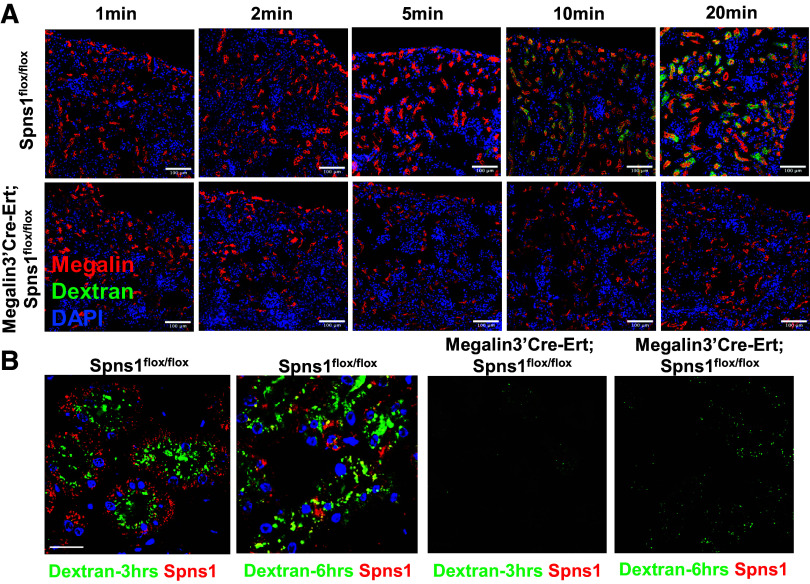
Conditional knockout of *Spns1* impairs proximal tubule endocytosis: time course. *A*: wild-type *(Spns1^flox/llox^*) and *Spns1*-deleted proximal tubules (*Megalin3’Cre-Ert;Spns1^flox/llox^*) could be distinguished within 10 min of infusion of FITC-dextran. *B*: dextran was captured by endosomes at the luminal surface and then subsequently located in SPNS1^+^ late endosomes and lysosomes deep in the cytoplasm. No labeling was found in *Spns1* knockouts. Bars = 100 µm in *A* and 20 µM in *B*.

The proximal tubule is critical for the capture of proteins that escape glomerular filtration barriers. To evaluate whether Spns1-mediated proteinuria was due to defective protein capture, we inoculated mice intraperitoneally with labeled proteins or with dextran and determined that *Spns1* deletion resulted in their excretion. Inoculated Alexa-labeled transferrin and Alexa-labeled NGAL were detected in the urine by visual inspection and by immunoblots (Fig. 4*C*). The capture of exogenous Alexa-NGAL, presumably by the megalin receptor (32), was defective (Fig. 4*D*). Even the capture of FITC-dextran, presumably by pinocytosis, was nearly abolished in *Megalin3’Cre-Ert;Spns1^flox/flox^* proximal tubules, despite qualitatively preserved levels of megalin staining (Fig. 4*E*). Time course experiments revealed that the reduction in proximal capture could be discerned within 10 min of FITC-dextran inoculation (Fig. 5*A*), suggesting disturbance in the initial stages of endocytosis. Consistent with this notion, defective endocytosis in *Spns1* deleted proximal tubules persisted when observing endosomal labeling with dextran over the course of many hours after the inoculation (Fig. 5*B*).

**Figure 6.**
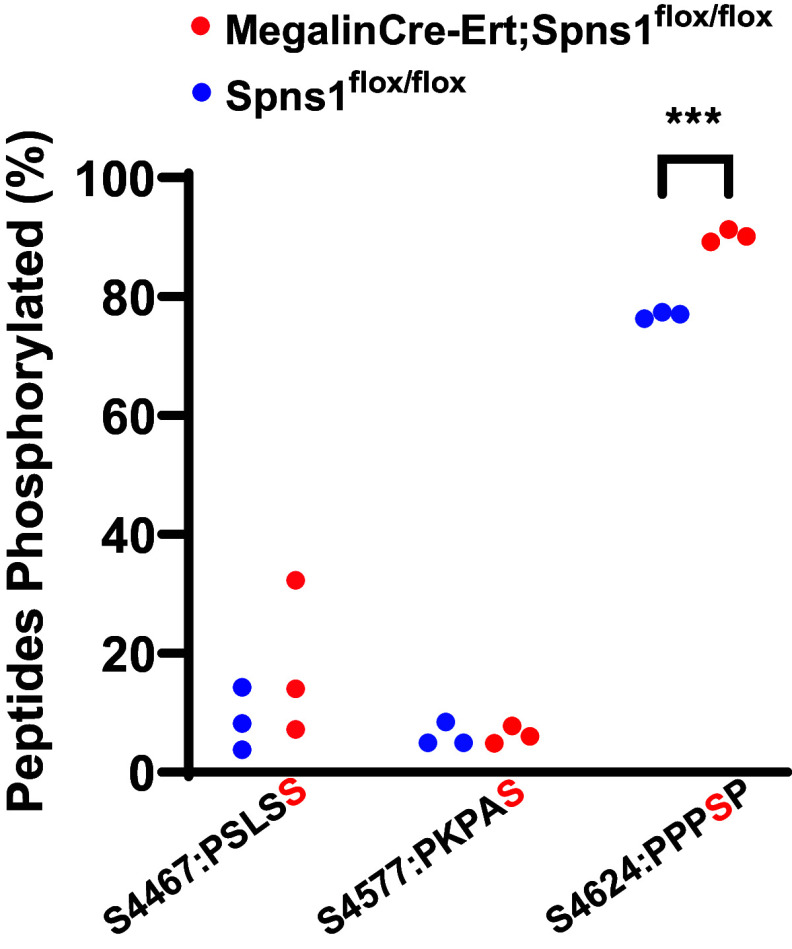
Conditional knockout of *Spns1* regulates phospho-megalin. *Megalin3’Cre-Ert;Spns1^flox/flox^* mice have a significant increase in phosphorylation at the PPPSP motif compared with *Spns1^flox/flox^* (***P = 2.79 × 10^−4^ by an unpaired two-tailed *t* test with Welch's correction; *n* = 3 mice each). Results from experiments in three separate mice yielded statistical significance. Phosphopeptides: S4467: KLPSLS**S**LAKPSENGNGVTFRS; S4577: R.SIDPSEIVPEPKPA**S**PGADETQGTK.W; and S4624: K.EAVAVAPPP**S**PSLPAKA.

**Figure 7.**
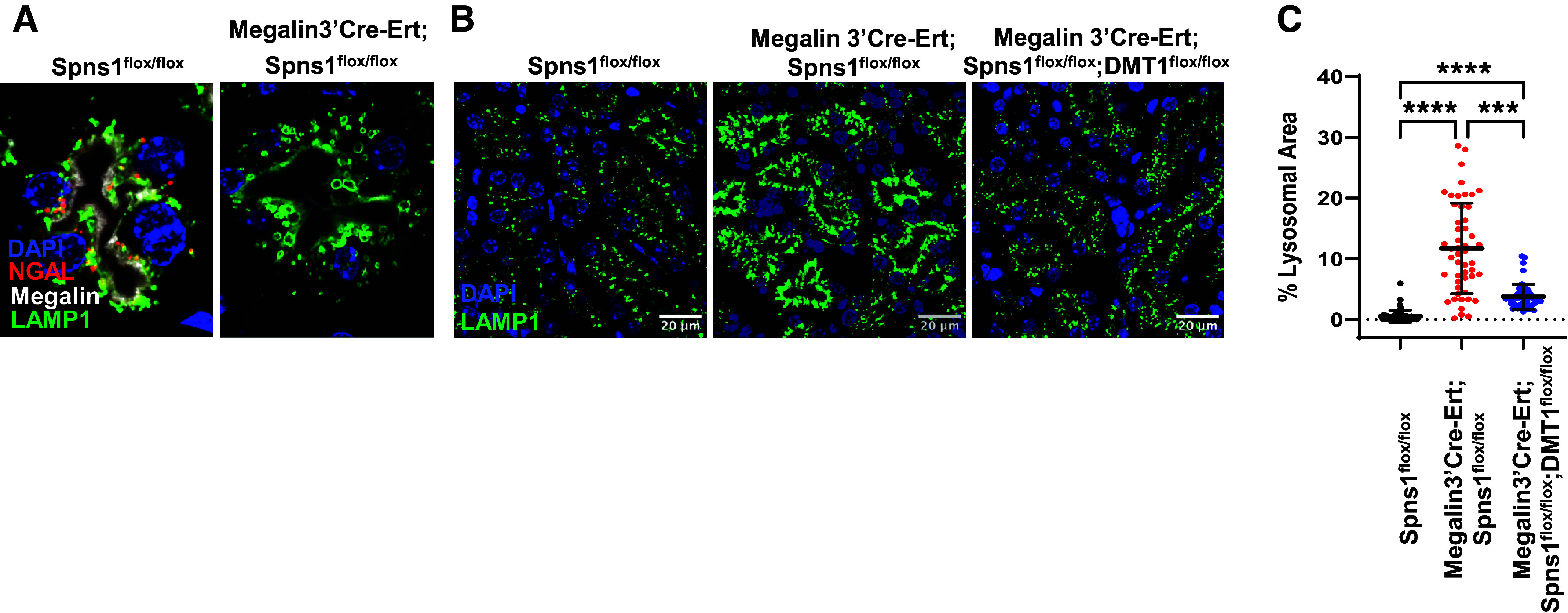
Conditional knockout of *Spns1* leads to endolysosomal swelling. *A*–*C*: LAMP1^+^ lysosomes doubled in size in *Megalin3’Cre-Ert;Spns1^flox/flox^* proximal tubules and occupied 22 times more area than lysosomes in *Spns1^flox/flox^* proximal tubules. The size of the lysosomes reverted to control size in *Megalin3’Cre-Ert;Spns1^flox/^^flox^;DMT1^flox/^^flox^* double knockout proximal tubules. *C* quantification of cytoplasmic area occupied by LAMP1^+^ lysosomes. Kruskal–Wallis with Dunn’s correction for multiple comparisons was used. ****WT vs. Spns1 KO, *P*_adj_ = 1.7 × 10^−23^; ****WT vs. Spns1KO;DMT1 KO, *P*_adj_ = 2.5 × 10^−9^; and ***Spns1KO vs. Spns1KO; DMT1 KO, *P*_adj_ = 2.2 × 10^−4^. Bars = 10 µm in *B*.

Additional evidence demonstrated that *Megalin3’Cre-Ert;Spns1^flox/flox^* induced dysfunction of many components of the endolysosomal pathways; phosphorylation of megalin motif PPPSP increased ∼16% (Fig. 6), implicating that the deletion of *Spns1* resulted in a change in megalin trafficking. In addition, lysosomes occupied ∼22-fold more area [reproducing published work (25, 29)] (Fig. 7, *A*–*C*).

### Conditional Knockout of Spns1 Leads to Iron Overload

**Figure 8.**
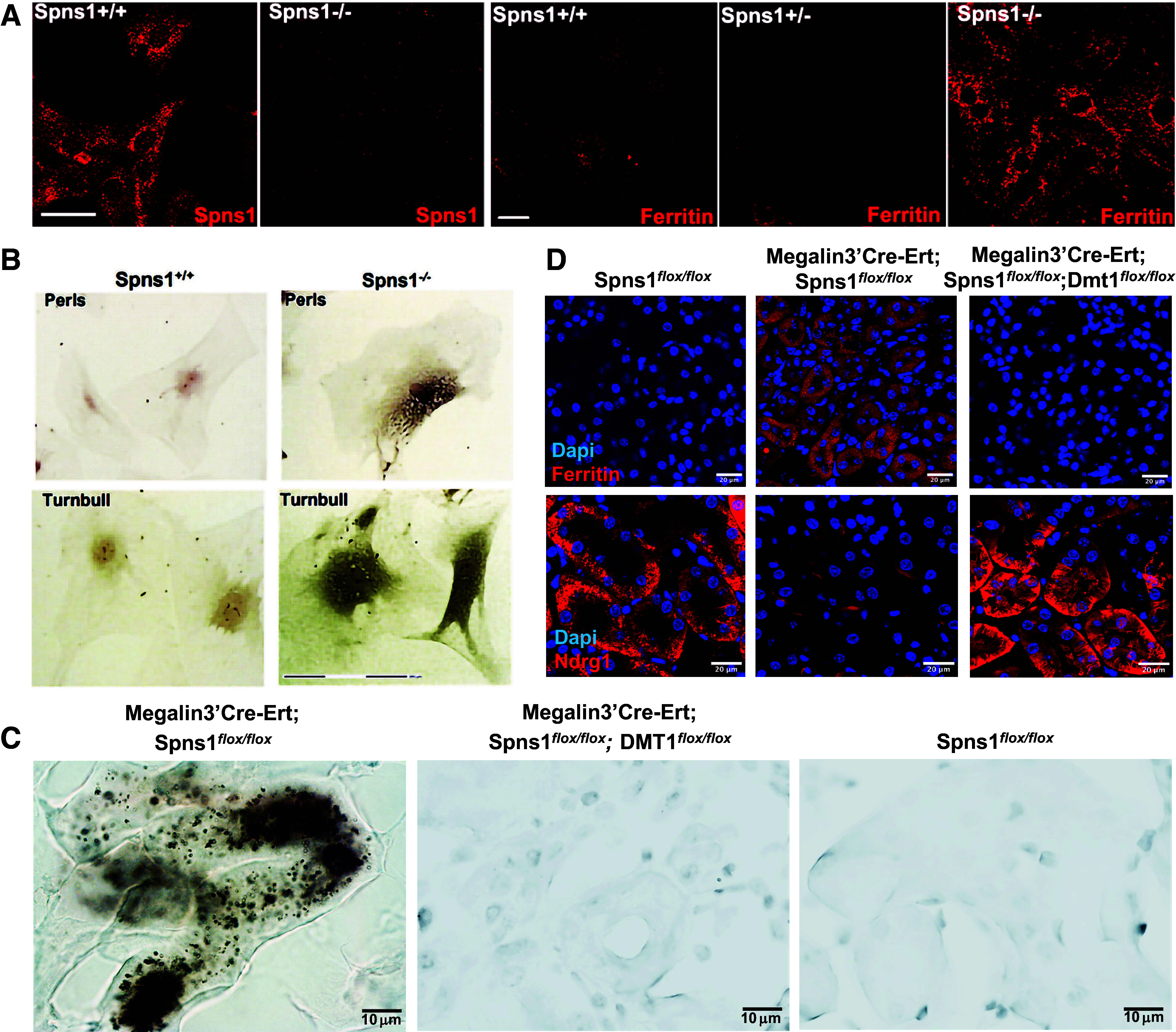
Conditional knockout of *Spns1* leads to iron overload. *A*: deletion of SPNS1 protein and accumulation of ferritin immunoreactivity in *Spns1*^−/−^ deleted MEFs. *B*: *Spns1*^−/−^ MEFs stain with Perls-DAB (*top* row) and Turnbull stains (*bottom* row) depicting Fe^3+^ or Fe^2+^ deposits, respectively. *C*: Perls-DAB staining of kidney sections. Increased staining in *Megalin3’Cre-Ert;Spns1^flox/flox^* kidneys was prevented by the additional deletion of *DMT1*. *D*: increased ferritin and decreased NDRG1 proteins in *Megalin3’Cre-Ert;Spns1^flox/flox^* knockout; there was reversion in *Megalin3’Cre-Ert;Spns1^flox/flox^;DMT1^flox/flox^* double knockouts. Bars = 20 µm in *A*, 150 µm in *B*, 10 µm in *C*, and 20 µm in D.

Given the evidence that *Spns1* expressing oocytes facilitate iron transport, we examined whether *Spns1* KO dysregulates cellular iron. We stained ferritin heavy chain (FTH1) in clones of MEFs derived from *Spns1*-gene trap mice (Fig. 8). These clones provide nonconditional deletion of *Spns1*, and the clones test cell-autonomous responses. *Spns1*^−/−^ MEF knockout clones demonstrated substantial loss of *Spns1* expression (Fig. 8*A*) (for example, expressing 0.584 + 0.112% of WT *Spns1* RNA); they exhibited increased cytoplasmic ferritin (Fig. 8*A*) as well as increased Perls-DAB (Fe^3+^) and Turnbull-DAB (Fe^2+^) staining (Fig. 8*B*), markers of cellular iron. Consistently, Perls-DAB staining (Fig. 8*C*) as well as FTH staining (Fig. 8*D*) was more prominent in the kidney cortex in *Megalin3’Cre-Ert*;*Spns1^flox/flox^* mice than in control mice. Ferritin and NDRG1 protein are expressed reciprocally and NDRG1 was depressed upon *Spns1* deletion (Fig. 8*D*).

Increased ferritin protein in tubular cells is consistent with iron inhibition of iron regulatory protein (IRP) binding to 5’-IRE in ferritin message (33). NDRG1 also responds to iron: elevated iron levels suppress eIF3a, which is crucial for the selective translation of *Ndrg1* mRNA (34). NDRG1, in turn, regulates cellular proliferation. These observations led us to hypothesize that *Spns1*-induced iron overload may alter mRNA transcript levels for a variety of iron-responsive genes.

To identify transcriptomic responses to *Spns1* deletion, we developed mice expressing Rosa26-floxed-stop-uracil phosphoribosyltransferase (*Uprt^flox/+^*), which drives tagging of nascent mRNA after inoculation with 4-thiouracil (19). We mated these mice with *Megalin3’Cre-Ert* driver mice so that nascent RNA was tagged specifically in the proximal tubule. We compared RNA from *Megalin3’Cre-Ert;Spns1^flox/^^flox^* with *Megalin3’Cre-Ert;Spns1^flox/+^* with and without iron overload (FeO, induced by three consecutive intraperitoneal injections of iron sucrose) to determine if similar genes were induced in the proximal tubule. PCA analysis demonstrated gross segregation of these data (Supplemental Fig. S1), but examination of each regulated gene (Supplemental Tables S2 and S3 and Supplemental Figs. S1–S4) demonstrated 42.9% of 3985 FeO significantly upregulated genes and 66.2% of 2582 *Spns1* significantly upregulated genes were common in the two models. Downregulated genes displayed a similar overlapping expression pattern. The data implicate iron overload as one component of the *Spns1* phenotype.

### Alleviation of Iron Overload Rescues Endocytosis in Spns1 Conditional Knockout Mice

**Figure 9.**
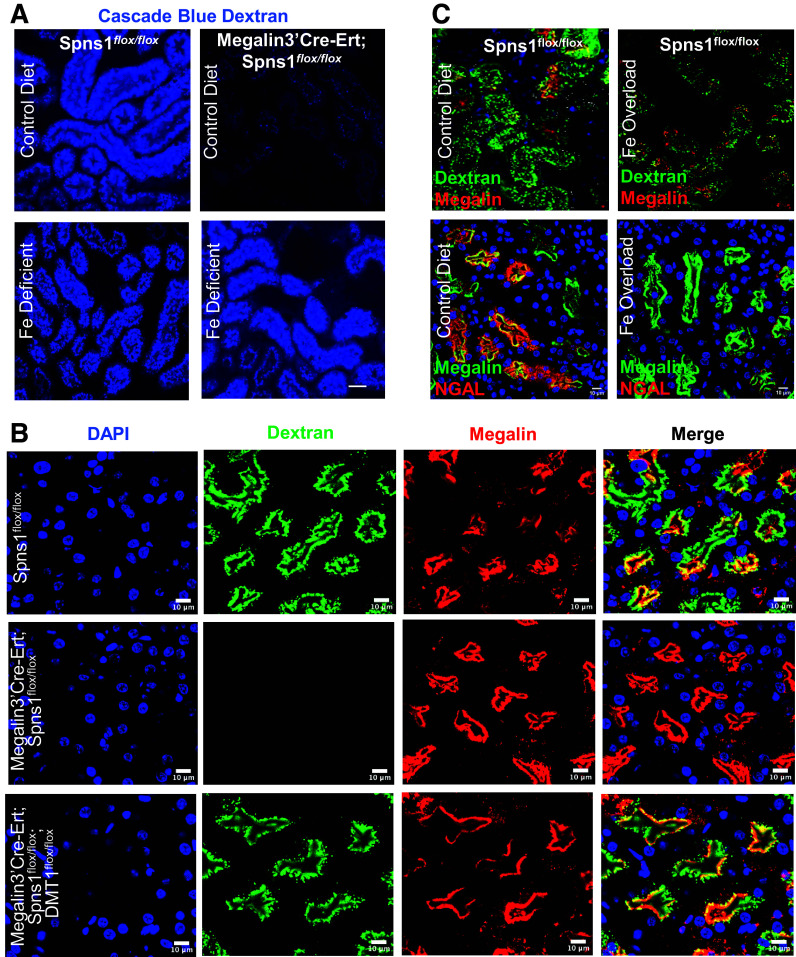
Iron-deficient chow or *DMT1* deletion rescues endocytosis. *A*: iron-deficient chow (*bottom* row) rescued the endocytosis of blue-dextran. *B:* likewise, double knockout *Megalin3’Cre-Ert;Spns1^flox/flox^;DMT1^flox/flox^* mice rescued endocytosis of FITC-dextran. *C*: conversely, iron overload of the kidney suppressed dextran and protein NGAL capture in wild-type kidneys. Bars = 25 µm in *A* and 10 µm in *B* and *C*.

To test whether iron overload might contribute to impaired endocytosis in *Spns1* KO mice, we used dietary and genetic methods to deplete iron from the kidney. First, we fed *Megalin3’Cre-Ert;Spns1^flox/flox^* mice either regular chow or iron-deficient chow for 6 wk coupled with three bleeds from the ophthalmic vein on consecutive weeks. This protocol reduced hematocrit to 33.2 ± 2.6% and, by inducing iron deficiency, rescued the capture of fluorescent dextran (Fig. 9*A*). To further investigate the association of iron load and endocytosis, we developed a double knockout, *Megalin3’Cre-Ert;Spns1^flox/^^flox^;DMT1^flox/^^flox^* mouse line that fully deleted *Spns1* and *DMT1* in the proximal tubule (*P*_adj_ = 0.013; Supplemental Fig. S5). Since DMT1 transports Fe^2+^ into the cell cytoplasm, we reasoned that knockdown of DMT1 would mitigate the Spns1 deletion. Indeed, knockout of DMT1 fully rescued the endocytic defect (Fig. 9*B*), the enlargement of lysosomes (Fig. 7, *A*, *B*, and *C*), and normalized ferritin and NDRG1 levels (Fig. 8*D*). Conversely, iron overload of wild-type mice inhibited both dextran and NGAL protein uptake in the kidney (Fig. 9*C*).

## DISCUSSION

Our data suggest that Spns1 regulates cellular iron. Its deletion produces an endocytic defect in the proximal tubule that is sensitive to dietary iron and DMT1 iron capture, implying an association between cellular iron and endocytosis. Iron is known to regulate a large number of genes at the transcriptional (35, 36), posttranscriptional [iron responsive elements (IRE) (37–39) and IRP (33, 40–43)], and enzymatic level (44). Analysis of RNA captured from the proximal tubule identified many cellular pathways that are affected by both *Spns1* deletion and iron overload (Supplemental Table S3), providing potential mechanisms for their association. Hence, the capture of the iron may activate feedback loops that, in turn, regulate apical endocytosis. In analogy, branched-chain amino acids and proteins captured by megalin regulate mTORC1 and, in turn, apical endocytosis (45).

*Spns1* deletion resulted in loss of endocytosis, implying that other Spns family members or other transporters cannot completely compensate for the deletion of *Spns1*. Nonetheless, although differences in Spns expression patterns in human and mouse kidneys have been documented, the Microdissected Mouse Kidney Tubule Database (46), Tabula Muris (47), and Susztak’s (48) and Humphrey’s (49) mouse databases demonstrate proximal tubule expression of the Spns family, raising the question of their interactions. Moreover, treatment of hepatoma cell lines with high doses of the iron chelator DFO (100 µM) upregulated *Spns2*, implying that iron deficiency might rescue the *Spns1* phenotype by inducing *Spns2* (50). To examine the issue of compensation directly, we performed RT-PCR on *Spns1^flox/^^flox^*, *Megalin3’Cre-Ert;Spns1^flox/^^flox^*, and *Megalin3’Cre-Ert;Spns1^flox/flox^*;*DMT1^flox/flox^* kidneys but failed to find gross changes in *Spns2* message that could explain the protective effect of DMT1 deletion. Likewise, iron restriction of *Spns1* knockouts did not induce *Spns2* expression while still reversing the endocytic block (Supplemental Fig. S6). In fact, to the contrary, both iron-overloaded proximal tubules and iron-rich *Spns1* deleted proximal tubules demonstrated slight increases in nascent *Spns2* message (Supplemental Table S2). We suggest that additional compound genetic knockouts will be needed to probe hierarchical relationships between Spns and other iron transporters in vivo.

The phenotype of *Spns1* deletion might be attributable to intrinsic changes in megalin. Megalin is the apical receptor that drives both protein capture as well as pinocytosis of dextrans. Receptor-mediated endocytosis results from 72 ligand binding repeats in the megalin dimer (23), while dextran pinocytosis is ascribed to megalin’s so-called “motor” function (51). Although gross changes in megalin expression or localization were not found, we identified a posttranslational modification, perhaps explaining a component of *Spns1*-mediated endocytic failure. Searching for phosphopeptides in purified apical membranes, we discovered increased serine phosphorylation at a PPPSP motif in megalin’s cytoplasmic tail in conditional *Spns1* KO relative to control (Fig. 6). This motif is a known regulator that shifts megalin from plasma to endocytic membranes (52). In short, nutrition affects posttranslational regulation of megalin, perhaps contributing to an endocytic defect.

Megalin phosphorylation may simply reflect a broader change in membrane dynamics. Counts from all expressed genes were used to identify enriched/deenriched gene sets using GSEA (Supplemental Table S3 and Supplemental Fig. S4*A*). As an example, both *Spns1* KO and iron-overloaded mice enriched the mTORC1 gene set and its component genes, including *Lamtor1*, −*2*, −*4*, and −*5*, Ragulator complex associated Rag GTPases A, C, and *Rheb*-GTP, a direct activator of the mTOR pathway (Supplemental Fig. S4*A*) (53). In addition, the inhibitors, *Tsc1*, the Rheb GAP, and *Prkaa1*, the α-AMPK subunit, were downregulated, perhaps prolonging mTORC1 activity (53, 54). Although these data might suggest enhancement of endocytosis (45), direct stimulation of S6K by purified mTORC1 as well as kidney immunoblots for p-S6K suggest dose-responsive inhibition of mTORC1 by iron (data not shown). Hence, in our model, mTORC1 is a candidate iron response pathway, but our data are currently indeterminant.

A second GSEA geneset of potential interest, highlighted by *Spns1* KO and iron overload or both, is a series of kinases and phosphatases that create the diversity of phoshoinositides (Supplemental Table S3 and Supplemental Fig. S4*B*). For example, plasma membrane PI(4,5)P_2_ recruits and assembles endocytic proteins (AP2 and clathrin) at the plasma membrane (55), but two upstream enzymes (PI4K2b and PIP5K1a) were downregulated in our models. Furthermore, the budding and release of endosomes requires dephosphorylation of PI(4,5)P_2_ (56), but its phosphatase, SYNJ1, is also downregulated, implying that endocytic failure is due to failure of phosphoinositide metabolism at the plasma membrane. Transcriptional data also suggested defects in clathrin-coated endosomal maturation, including SNX9-mediated membrane constriction at the plasma membrane because essential enzymes PI4K2B, PI3KC2a, and INPP4a (57–59) are significantly suppressed by our *Spns1* KO or iron overload model (Supplemental Table S2). For example, the reduction of PI(3)P may inhibit the recruitment of trafficking proteins such as early endosome autoantigen 1 (EEA1) and ESCRTs and cargo sorting at the membrane of late endosomes due to downregulation of enzyme PIKFYVE (57). In summary, transcriptional data (Supplemental Table S2) depicted the suppression of an enzyme cascade giving rise to phoshoinositides, consistent with a *Spns1*-mediated endocytic defect. These data offer new avenues to explore the intersection of iron and membrane trafficking. This hypothesis is compatible with published data from *Drosophila*, *C. elegans*, and murine cell lines, showing disruption of membrane flows and autophagy in *Spns1* knockouts (29, 60–63).

It remains possible that endolysosomal dysfunction is primary and induces iron loading, perhaps by modifying DMT1, TfR1, and ferritin traffic. Yet exogenous iron load suppressed endocytosis and mimicked *Spns1* KO phenotype, while iron deficiency or deletion of DMT1 reversed the failure of endocytosis. In addition, the directionality of the *Spns1* channel remains to be established, but the accumulation of cytoplasmic ferritin and the rescue of endocytosis by deletion of DMT1 (Fig. 9*B*) suggest that *Spns1* may function to export iron from the cytoplasm. If this is the case, then additional combinatorial knockouts may enhance (*Spns1* + *Fpn*) or suppress (*Spns1* + *TfR1*) the endocytic defect and determine Spns1’s role in the hierarchy of these other iron transporters.

Landmark discoveries demonstrate that iron capture is directly regulated by its substrate iron. *TfR1* is directly regulated by iron-sensitive 3′-UTR iron response elements (43, 64), while ferroportin protein trafficking is directly regulated by hepcidin (65). Here we show that cellular iron might also control iron scavenging by regulating endocytosis in the kidney. The type and strength of the feedback depends not only on cellular iron distribution but also on how the proximal tubule obtains iron. These inputs can change over the course of kidney development and kidney damage, where TfR1 and megalin have evolving patterns of expression. Hence, the regulation of iron capture pathways will determine feedback to megalin-dependent endocytosis, which is best investigated by combinatorial knockouts.

## DATA AVAILABILITY

The raw mass spectrometry data in this study are available at MassIVE (UCSD, https://massive.ucsd.edu/ProteoSAFe/static/massive.jsp) under Accession No. MSV000094931 (https://massive.ucsd.edu/ProteoSAFe/dataset.jsp?task=b8c19ecdb1d94b15906cc2bc773a4f45) and the Gene Expression Omnibus (series GSE268837).

## GRANTS

This work was supported by National Institutes of Health (NIH) Grants R01DK124667 and U54DK104309 (to J.B.), K08DK132511 (to A.B.), K25DK128563 (to A.K.) and 1S10RR027990 (to T.A.N.) as well as China National Science Foundation Grants 31271551 and 81970593 (to A.Q.). A.B. received additional support from the Gerstner Family Foundation and an American Society of Nephrology and Kidney Cure Carl W. Gottschalk Research Scholar Award. This research was funded in part through the NIH Grant P30CA013696 and used the Genomics and High Throughput Screening Shared Resource.

## DISCLOSURES

No conflicts of interest, financial or otherwise, are declared by the authors.

## AUTHOR CONTRIBUTIONS

A.Q. and J.B. conceived and designed research; A.B., T.S., G.J., A.G., K.X., S.V., A.L., E.Y.C., S.Y.R.-J., L.H., T.B.F., H.E.-B., A.Q., and J.B. performed experiments; A.B., T.S., G.J., A.G., K.X., K.N., R.E.S., S.V., A.K., A.L., J.S., E.Y.C., S.Y.R.-J., H.E.-B., T.A.N., A.Q., and J.B. analyzed data; A.B., T.S., G.J., A.G., K.X., K.N., R.E.S., S.V., A.L., J.S., E.Y.C., S.Y.R.-J., H.E.-B., T.A.N., L.S., A.Q., and J.B. interpreted results of experiments; A.B., T.S., G.J., A.G., K.X., A.Q., and J.B. prepared figures; A.B., A.Q., and J.B. drafted the manuscript; A.B., T.S., K.X., A.Q., and J.B. edited and revised the manuscript; A.B., T.S., G.J., A.G., K.X., K.N., R.E.S., S.V., A.K., A.L., J.S., E.Y.C., S.Y.R.-J., L.H., T.B.F., H.E.-B., T.A.N., L.S., A.Q., and J.B. approved final version of manuscript.

## References

[1] Nielsen R, Christensen EI, Birn H. Megalin and cubilin in proximal tubule protein reabsorption: from experimental models to human disease. Kidney Int 89: 58–67, 2016. doi:10.1016/j.kint.2015.11.007. 26759048

[2] Lieu PT, Heiskala M, Peterson PA, Yang Y. The roles of iron in health and disease. Mol Aspects Med 22: 1–87, 2001. doi:10.1016/s0098-2997(00)00006-6. 11207374

[3] Ponka P. Cellular iron metabolism. Kidney Int Suppl 69: S2–S11, 1999. doi:10.1046/j.1523-1755.1999.055suppl.69002.x. 10084280

[4] Ponka P, Lok CN. The transferrin receptor: role in health and disease. Int J Biochem Cell Biol 31: 1111–1137, 1999. doi:10.1016/s1357-2725(99)00070-9. 10582342

[5] Aisen P. Transferrin receptor 1. Int J Biochem Cell Biol 36: 2137–2143, 2004. doi:10.1016/j.biocel.2004.02.007. 15313461

[6] Lamb JE, Ray F, Ward JH, Kushner JP, Kaplan J. Internalization and subcellular localization of transferrin and transferrin receptors in HeLa cells. J Biol Chem 258: 8751–8758, 1983. 6305999

[7] McKie AT, Barrow D, Latunde-Dada GO, Rolfs A, Sager G, Mudaly E, Mudaly M, Richardson C, Barlow D, Bomford A, Peters TJ, Raja KB, Shirali S, Hediger MA, Farzaneh F, Simpson RJ. An iron-regulated ferric reductase associated with the absorption of dietary iron. Science 291: 1755–1759, 2001. doi:10.1126/science.1057206. 11230685

[8] Hamill RL, Woods JC, Cook BA. Congenital atransferrinemia. A case report and review of the literature. Am J Clin Pathol 96: 215–218, 1991. doi:10.1093/ajcp/96.2.215. 1862777

[9] Hayashi A, Wada Y, Suzuki T, Shimizu A. Studies on familial hypotransferrinemia: unique clinical course and molecular pathology. Am J Hum Genet 53: 201–213, 1993. 8317485 PMC1682235

[10] Huggenvik JI, Craven CM, Idzerda RL, Bernstein S, Kaplan J, McKnight GS. A splicing defect in the mouse transferrin gene leads to congenital atransferrinemia. Blood 74: 482–486, 1989. 2752125

[11] Levy JE, Jin O, Fujiwara Y, Kuo F, Andrews NC. Transferrin receptor is necessary for development of erythrocytes and the nervous system. Nat Genet 21: 396–399, 1999. doi:10.1038/7727. 10192390

[12] Li JY, Paragas N, Ned RM, Qiu A, Viltard M, Leete T, Drexler IR, Chen X, Sanna-Cherchi S, Mohammed F, Williams D, Lin CS, Schmidt-Ott KM, Andrews NC, Barasch J. Scara5 is a ferritin receptor mediating non-transferrin iron delivery. Dev Cell 16: 35–46, 2009. doi:10.1016/j.devcel.2008.12.002. 19154717 PMC2652503

[13] Ned RM, Swat W, Andrews NC. Transferrin receptor 1 is differentially required in lymphocyte development. Blood 102: 3711–3718, 2003. doi:10.1182/blood-2003-04-1086. 12881306

[14] Trenor CC, 3rd, Campagna DR, Sellers VM, Andrews NC, Fleming MD. The molecular defect in hypotransferrinemic mice. Blood 96: 1113–1118, 2000. 10910930

[15] Ha HT, Liu S, Nguyen XT, Vo LK, Leong NC, Nguyen DT, Balamurugan S, Lim PY, Wu Y, Seong E, Nguyen TQ, Oh J, Wenk MR, Cazenave-Gassiot A, Yapici Z, Ong WY, Burmeister M, Nguyen LN. Lack of SPNS1 results in accumulation of lysolipids and lysosomal storage disease in mouse models. JCI Insight 9: e175462, 2024. doi:10.1172/jci.insight.175462. 38451736 PMC11141868

[16] He M, Kuk ACY, Ding M, Chin CF, Galam DLA, Nah JM, Tan BC, Yeo HL, Chua GL, Benke PI, Wenk MR, Ho L, Torta F, Silver DL. Spns1 is a lysophospholipid transporter mediating lysosomal phospholipid salvage. Proc Natl Acad Sci USA 119: e2210353119, 2022. doi:10.1073/pnas.2210353119. 36161949 PMC9546575

[17] Scharenberg SG, Dong W, Ghoochani A, Nyame K, Levin-Konigsberg R, Krishnan AR, Rawat ES, Spees K, Bassik MC, Abu-Remaileh M. An SPNS1-dependent lysosomal lipid transport pathway that enables cell survival under choline limitation. Sci Adv 9: eadf8966, 2023. doi:10.1126/sciadv.adf8966. 37075117 PMC10115416

[18] Qiu A, Jansen M, Sakaris A, Min SH, Chattopadhyay S, Tsai E, Sandoval C, Zhao R, Akabas MH, Goldman ID. Identification of an intestinal folate transporter and the molecular basis for hereditary folate malabsorption. Cell 127: 917–928, 2006. doi:10.1016/j.cell.2006.09.041. 17129779

[19] Shen TH, Stauber J, Xu K, Jacunski A, Paragas N, Callahan M, Banlengchit R, Levitman AD, Desanti De Oliveira B, Beenken A, Grau MS, Mathieu E, Zhang Q, Li Y, Gopal T, Askanase N, Arumugam S, Mohan S, Good PI, Stevens JS, Lin F, Sia SK, Lin CS, D'Agati V, Kiryluk K, Tatonetti NP, Barasch J. Snapshots of nascent RNA reveal cell- and stimulus-specific responses to acute kidney injury. JCI Insight 7: e146374, 2022. doi:10.1172/jci.insight.146374. 35230973 PMC8986083

[20] Meguro R, Asano Y, Odagiri S, Li C, Iwatsuki H, Shoumura K. The presence of ferric and ferrous iron in the nonheme iron store of resident macrophages in different tissues and organs: histochemical demonstrations by the perfusion-Perls and -Turnbull methods in the rat. Arch Histol Cytol 68: 171–183, 2005. doi:10.1679/aohc.68.171. 16276023

[21] Nguyen-Legros J, Bizot J, Bolesse M, Pulicani JP. [“Diaminobenzidine black” as a new histochemical demonstration of exogenous iron (author's transl)]. Histochemistry 66: 239–244, 1980. doi:10.1007/BF00495737. 7399970

[22] Carson FL. Histotechnology: a Self Instructional Text (5th ed.). Chicago, IL: ASCP Press, American Society of Clinical Pathologists, 1990.

[23] Beenken A, Cerutti G, Brasch J, Guo Y, Sheng Z, Erdjument-Bromage H, Aziz Z, Robbins-Juarez SY, Chavez EY, Ahlsen G, Katsamba PS, Neubert TA, Fitzpatrick AWP, Barasch J, Shapiro L. Structures of LRP2 reveal a molecular machine for endocytosis. Cell 186: 821–836.e13, 2023. doi:10.1016/j.cell.2023.01.016. 36750096 PMC9993842

[24] Sweeney ST, Davis GW. Unrestricted synaptic growth in spinster-a late endosomal protein implicated in TGF-beta-mediated synaptic growth regulation. Neuron 36: 403–416, 2002. doi:10.1016/s0896-6273(02)01014-0. 12408844

[25] Dermaut B, Norga KK, Kania A, Verstreken P, Pan H, Zhou Y, Callaerts P, Bellen HJ. Aberrant lysosomal carbohydrate storage accompanies endocytic defects and neurodegeneration in *Drosophila* benchwarmer. J Cell Biol 170: 127–139, 2005. doi:10.1083/jcb.200412001. PMC2171373 15998804

[26] Nakano Y, Fujitani K, Kurihara J, Ragan J, Usui-Aoki K, Shimoda L, Lukacsovich T, Suzuki K, Sezaki M, Sano Y, Ueda R, Awano W, Kaneda M, Umeda M, Yamamoto D. Mutations in the novel membrane protein spinster interfere with programmed cell death and cause neural degeneration in *Drosophila melanogaster*. Mol Cell Biol 21: 3775–3788, 2001. doi:10.1128/MCB.21.11.3775-3788.2001. 11340170 PMC87027

[27] Usui-Aoki K, Nakano Y, Yamamoto D. Pathology of the adult central nervous system induced by genetic inhibition of programmed cell death in *Drosophila* pupae. Arch Insect Biochem Physiol 49: 94–101, 2002 [Erratum in *Arch Insect Biochem Physiol* 49: 225–226, 2002]. doi:10.1002/arch.10011. 11816024

[28] Young RM, Marty S, Nakano Y, Wang H, Yamamoto D, Lin S, Allende ML. Zebrafish yolk-specific not really started (nrs) gene is a vertebrate homolog of the *Drosophila* spinster gene and is essential for embryogenesis. Dev Dyn 223: 298–305, 2002. doi:10.1002/dvdy.10060. 11836794

[29] Rong Y, McPhee CK, Deng S, Huang L, Chen L, Liu M, Tracy K, Baehrecke EH, Yu L, Lenardo MJ. Spinster is required for autophagic lysosome reformation and mTOR reactivation following starvation. Proc Natl Acad Sci USA 108: 7826–7831, 2011 [Erratum in *Proc Natl Acad Sci USA* 108: 11297, 2011]. doi:10.1073/pnas.1013800108. 21518918 PMC3093520

[30] Yanagisawa H, Miyashita T, Nakano Y, Yamamoto D. HSpin1, a transmembrane protein interacting with Bcl-2/Bcl-xL, induces a caspase-independent autophagic cell death. Cell Death Differ 10: 798–807, 2003. doi:10.1038/sj.cdd.4401246. 12815463

[31] Zhao M, Li M, Yang Y, Guo Z, Sun Y, Shao C, Li M, Sun W, Gao Y. A comprehensive analysis and annotation of human normal urinary proteome. Sci Rep 7: 3024, 2017. doi:10.1038/s41598-017-03226-6. 28596590 PMC5465101

[32] Barasch J, Hollmen M, Deng R, Hod EA, Rupert PB, Abergel RJ, Allred BE, Xu K, Darrah SF, Tekabe Y, Perlstein A, Wax R, Bruck E, Stauber J, Corbin KA, Buchen C, Slavkovich V, Graziano J, Spitalnik SL, Bao G, Strong RK, Qiu A. Disposal of iron by a mutant form of lipocalin 2. Nat Commun 7: 12973, 2016. doi:10.1038/ncomms12973. 27796299 PMC5095531

[33] Eisenstein RS, Blemings KP. Iron regulatory proteins, iron responsive elements and iron homeostasis. J Nutr 128: 2295–2298, 1998. doi:10.1093/jn/128.12.2295. 9868172

[34] Lane DJ, Saletta F, Suryo Rahmanto Y, Kovacevic Z, Richardson DR. N-myc downstream regulated 1 (NDRG1) is regulated by eukaryotic initiation factor 3a (eIF3a) during cellular stress caused by iron depletion.PLoS One 8: e57273, 2013. doi:10.1371/journal.pone.0057273. 23437357 PMC3578820

[35] Gazitt Y, Reddy SV, Alcantara O, Yang J, Boldt DH. A new molecular role for iron in regulation of cell cycling and differentiation of HL-60 human leukemia cells: iron is required for transcription of p21(WAF1/CIP1) in cells induced by phorbol myristate acetate. J Cell Physiol 187: 124–135, 2001. doi:10.1002/1097-4652(2001)9999:9999<::AID-JCP1061>3.0.CO;2-E. 11241357

[36] Ye Z, Connor JR. Screening of transcriptionally regulated genes following iron chelation in human astrocytoma cells. Biochem Biophys Res Commun 264: 709–713, 1999. doi:10.1006/bbrc.1999.1554. 10543996

[37] Aziz N, Munro HN. Iron regulates ferritin mRNA translation through a segment of its 5' untranslated region. Proc Natl Acad Sci USA 84: 8478–8482, 1987. doi:10.1073/pnas.84.23.8478. 3479802 PMC299567

[38] Rouault TA. Post-transcriptional regulation of human iron metabolism by iron regulatory proteins. Blood Cells Mol Dis 29: 309–314, 2002. doi:10.1006/bcmd.2002.0571. 12547221

[39] Haile DJ. Regulation of genes of iron metabolism by the iron-response proteins. Am J Med Sci 318: 230–240, 1999. doi:10.1097/00000441-199910000-00003. 10522551

[40] Henderson BR, Kuhn LC. Differential modulation of the RNA-binding proteins IRP-1 and IRP-2 in response to iron. IRP-2 inactivation requires translation of another protein. J Biol Chem 270: 20509–20515, 1995. doi:10.1074/jbc.270.35.20509. 7544791

[41] LaVaute T, Smith S, Cooperman S, Iwai K, Land W, Meyron-Holtz E, Drake SK, Miller G, Abu-Asab M, Tsokos M, Switzer R 3rd, Grinberg A, Love P, Tresser N, Rouault TA. Targeted deletion of the gene encoding iron regulatory protein-2 causes misregulation of iron metabolism and neurodegenerative disease in mice. Nat Genet 27: 209–214, 2001. doi:10.1038/84859. 11175792

[42] Leibold EA, Munro HN. Cytoplasmic protein binds in vitro to a highly conserved sequence in the 5' untranslated region of ferritin heavy- and light-subunit mRNAs. Proc Natl Acad Sci U S A 85: 2171–2175, 1988. doi:10.1073/pnas.85.7.2171. 3127826 PMC279951

[43] Rouault TA, Hentze MW, Caughman SW, Harford JB, Klausner RD. Binding of a cytosolic protein to the iron-responsive element of human ferritin messenger RNA. Science 241: 1207–1210, 1988. doi:10.1126/science.3413484. 3413484

[44] Cooper CE, Lynagh GR, Hoyes KP, Hider RC, Cammack R, Porter JB. The relationship of intracellular iron chelation to the inhibition and regeneration of human ribonucleotide reductase. J Biol Chem 271: 20291–20299, 1996. doi:10.1074/jbc.271.34.20291. 8702762

[45] Grahammer F, Ramakrishnan SK, Rinschen MM, Larionov AA, Syed M, Khatib H, Roerden M, Sass JO, Helmstaedter M, Osenberg D, Kuhne L, Kretz O, Wanner N, Jouret F, Benzing T, Artunc F, Huber TB, Theilig F. mTOR regulates endocytosis and nutrient transport in proximal tubular cells. J Am Soc Nephrol 28: 230–241, 2017. doi:10.1681/ASN.2015111224. 27297946 PMC5198276

[46] Chen L, Chou CL, Knepper MA. A Comprehensive map of mRNAs and their isoforms across all 14 renal tubule segments of mouse. J Am Soc Nephrol 32: 897–912, 2021. doi:10.1681/ASN.2020101406. 33769951 PMC8017530

[47] Tabula Muris Consortium, Overall coordination, Logistical coordination, Organ collection and processing, Library preparation and sequencing, Computational data analysis, Cell type annotation, Writing group, Supplemental text writing group, and Principal investigators. Single-cell transcriptomics of 20 mouse organs creates a Tabula Muris. Nature 562: 367–372, 2018. 30283141 10.1038/s41586-018-0590-4 PMC6642641

[48] Balzer MS, Doke T, Yang YW, Aldridge DL, Hu H, Mai H, Mukhi D, Ma Z, Shrestha R, Palmer MB, Hunter CA, Susztak K. Single-cell analysis highlights differences in druggable pathways underlying adaptive or fibrotic kidney regeneration. Nat Commun 13: 4018, 2022. doi:10.1038/s41467-022-31772-9. 35821371 PMC9276703

[49] Wu H, Kirita Y, Donnelly EL, Humphreys BD. Advantages of single-nucleus over single-cell RNA sequencing of adult kidney: rare cell types and novel cell states revealed in fibrosis. J Am Soc Nephrol 30: 23–32, 2019. doi:10.1681/ASN.2018090912. 30510133 PMC6317600

[50] Wang D, Li M, Shen H, Yang J, Gao Z, Tang Y. Iron deficiency increases phosphorylation of SP1 to upregulate SPNS2 expression in hepatocellular carcinoma. Biol Trace Elem Res 201: 1689–1694, 2023. doi:10.1007/s12011-022-03296-2. 35614326

[51] Rbaibi Y, Long KR, Shipman KE, Ren Q, Baty CJ, Kashlan OB, Weisz OA. Megalin, cubilin, and Dab2 drive endocytic flux in kidney proximal tubule cells. Mol Biol Cell 34: ar74, 2023. doi:10.1091/mbc.E22-11-0510. 37126375 PMC10295476

[52] Yuseff MI, Farfan P, Bu G, Marzolo MP. A cytoplasmic PPPSP motif determines megalin's phosphorylation and regulates receptor's recycling and surface expression. Traffic 8: 1215–1230, 2007. doi:10.1111/j.1600-0854.2007.00601.x. 17555532

[53] Fernandes SA, Demetriades C. The multifaceted role of nutrient sensing and mTORC1 signaling in physiology and aging. Front Aging 2: 707372, 2021. doi:10.3389/fragi.2021.707372. 35822019 PMC9261424

[54] Shang C, Zhou H, Liu W, Shen T, Luo Y, Huang S. Iron chelation inhibits mTORC1 signaling involving activation of AMPK and REDD1/Bnip3 pathways. Oncogene 39: 5201–5213, 2020. doi:10.1038/s41388-020-1366-5. 32541839 PMC7366895

[55] van den Bout I, Divecha N. PIP5K-driven PtdIns(4,5)P2 synthesis: regulation and cellular functions. J Cell Sci 122: 3837–3850, 2009. doi:10.1242/jcs.056127. 19889969

[56] Fasano D, Parisi S, Pierantoni GM, De Rosa A, Picillo M, Amodio G, Pellecchia MT, Barone P, Moltedo O, Bonifati V, De Michele G, Nitsch L, Remondelli P, Criscuolo C, Paladino S. Alteration of endosomal trafficking is associated with early-onset parkinsonism caused by SYNJ1 mutations. Cell Death Dis 9: 385, 2018. doi:10.1038/s41419-018-0410-7. 29515184 PMC5841278

[57] Posor Y, Eichhorn-Gruenig M, Puchkov D, Schoneberg J, Ullrich A, Lampe A, Muller R, Zarbakhsh S, Gulluni F, Hirsch E, Krauss M, Schultz C, Schmoranzer J, Noe F, Haucke V. Spatiotemporal control of endocytosis by phosphatidylinositol-3,4-bisphosphate. Nature 499: 233–237, 2013. doi:10.1038/nature12360. 23823722

[58] Shin HW, Hayashi M, Christoforidis S, Lacas-Gervais S, Hoepfner S, Wenk MR, Modregger J, Uttenweiler-Joseph S, Wilm M, Nystuen A, Frankel WN, Solimena M, De Camilli P, Zerial M. An enzymatic cascade of Rab5 effectors regulates phosphoinositide turnover in the endocytic pathway. J Cell Biol 170: 607–618, 2005. doi:10.1083/jcb.200505128. 16103228 PMC2171494

[59] Wallroth A, Haucke V. Phosphoinositide conversion in endocytosis and the endolysosomal system. J Biol Chem 293: 1526–1535, 2018. doi:10.1074/jbc.R117.000629. 29282290 PMC5798284

[60] Sakurai A, Nakano Y, Koganezawa M, Yamamoto D. Phenotypic interactions of spinster with the genes encoding proteins for cell death control in Drosophila melanogaster. Arch Insect Biochem Physiol 73: 119–127, 2010. doi:10.1002/arch.20345. 20091795

[61] Sasaki T, Lian S, Khan A, Llop JR, Samuelson AV, Chen W, Klionsky DJ, Kishi S. Autolysosome biogenesis and developmental senescence are regulated by both Spns1 and v-ATPase. Autophagy 13: 386–403, 2017. doi:10.1080/15548627.2016.1256934. 27875093 PMC5324850

[62] Sasaki T, Lian S, Qi J, Bayliss PE, Carr CE, Johnson JL, Guha S, Kobler P, Catz SD, Gill M, Jia K, Klionsky DJ, Kishi S. Aberrant autolysosomal regulation is linked to the induction of embryonic senescence: differential roles of Beclin 1 and p53 in vertebrate Spns1 deficiency. PLoS Genet 10: e1004409, 2014. doi:10.1371/journal.pgen.1004409. 24967584 PMC4072523

[63] Yanagisawa H, Ishii T, Endo K, Kawakami E, Nagao K, Miyashita T, Akiyama K, Watabe K, Komatsu M, Yamamoto D, Eto Y. L-leucine and SPNS1 coordinately ameliorate dysfunction of autophagy in mouse and human Niemann-Pick type C disease. Sci Rep 7: 15944, 2017. doi:10.1038/s41598-017-15305-9. 29162837 PMC5698481

[64] Hentze MW, Caughman SW, Rouault TA, Barriocanal JG, Dancis A, Harford JB, Klausner RD. Identification of the iron-responsive element for the translational regulation of human ferritin mRNA. Science 238: 1570–1573, 1987. doi:10.1126/science.3685996. 3685996

[65] Nemeth E, Tuttle MS, Powelson J, Vaughn MB, Donovan A, Ward DM, Ganz T, Kaplan J. Hepcidin regulates cellular iron efflux by binding to ferroportin and inducing its internalization. Science 306: 2090–2093, 2004. doi:10.1126/science.1104742. 15514116

